# Climatic and edaphic controls over tropical forest diversity and vegetation carbon storage

**DOI:** 10.1038/s41598-020-61868-5

**Published:** 2020-03-19

**Authors:** Florian Hofhansl, Eduardo Chacón-Madrigal, Lucia Fuchslueger, Daniel Jenking, Albert Morera-Beita, Christoph Plutzar, Fernando Silla, Kelly M. Andersen, David M. Buchs, Stefan Dullinger, Konrad Fiedler, Oskar Franklin, Peter Hietz, Werner Huber, Carlos A. Quesada, Anja Rammig, Franziska Schrodt, Andrea G. Vincent, Anton Weissenhofer, Wolfgang Wanek

**Affiliations:** 1 International Institute for Applied Systems Analysis, Schlossplatz 1, A-2361 Laxenburg, Austria; 2 Escuela de Biología, Universidad de Costa Rica, San José, Costa Rica; 3 Department of Biology, Plants and Ecosystems, University of Antwerp, Antwerp, Belgium; 4 Escuela de Agronomía, Universidad de Costa Rica, San José, Costa Rica; 5 Laboratory of Applied Tropical Ecology, National University of Costa Rica, Heredia, Costa Rica; 6 Department of Botany & Biodiversity Research, University of Vienna, Vienna, Austria; 7 Institute of Social Ecology, University of Natural Resources and Life Sciences, Vienna, Austria; 8 Area of Ecology, Faculty of Biology, University of Salamanca, Salamanca, Spain; 9 Nanyang Technological University, Asian School of the Environment, 50 Nanyang Avenue, 639798 Singapore, Singapore; 10 School of Earth and Ocean Sciences, Cardiff University, Park Place, Cardiff CF10 3AT UK; 11 Department of Integrative Biology and Biodiversity Research, Institute of Botany, University of Natural Resources and Life Sciences, Vienna, Austria; 12 Instituto Nacional de Pesquisas da Amazônia, Coordenação de Dinâmica Ambiental, Avenida Ephigenio Salles 2239, Aleixo - 69000000, Manaus, AM Brasil; 13 Technical University of Munich, TUM School of Life Sciences Weihenstephan, Hans-Carl-v.-Carlowitz-Platz 2, 85354 Freising, Germany; 14 School of Geography, University of Nottingham, University Park, Nottingham, NG7 2RD United Kingdom; 15 Department of Microbiology & Ecosystem Science, Division of Terrestrial Ecosystem Research, University of Vienna, Vienna, Austria

**Keywords:** Forest ecology, Carbon cycle, Biodiversity

## Abstract

Tropical rainforests harbor exceptionally high biodiversity and store large amounts of carbon in vegetation biomass. However, regional variation in plant species richness and vegetation carbon stock can be substantial, and may be related to the heterogeneity of topoedaphic properties. Therefore, aboveground vegetation carbon storage typically differs between geographic forest regions in association with the locally dominant plant functional group. A better understanding of the underlying factors controlling tropical forest diversity and vegetation carbon storage could be critical for predicting tropical carbon sink strength in response to projected climate change. Based on regionally replicated 1-ha forest inventory plots established in a region of high geomorphological heterogeneity we investigated how climatic and edaphic factors affect tropical forest diversity and vegetation carbon storage. Plant species richness (of all living stems >10 cm in diameter) ranged from 69 to 127 ha^−1^ and vegetation carbon storage ranged from 114 to 200 t ha^−1^. While plant species richness was controlled by climate and soil water availability, vegetation carbon storage was strongly related to wood density and soil phosphorus availability. Results suggest that local heterogeneity in resource availability and plant functional composition should be considered to improve projections of tropical forest ecosystem functioning under future scenarios.

## Introduction

Tropical forests host two thirds of terrestrial biota^1^ and comprise one fourth of the planet’s terrestrial carbon (C) stored in aboveground vegetation biomass (AGB)^2^. It has been proposed that biodiversity positively affects carbon storage in hyper-diverse tropical forests^3^, but this finding has been repeatedly challenged by studies showing that relationships between species diversity and ecosystem functioning are dependent on the scale of observation^4,5^, and usually saturate at high levels of species richness, such as in tropical forests^6,7^. As a consequence, relationships between biodiversity and C storage remain poorly resolved for tropical forests^6–8^. It is inherently difficult to disentangle factors determining tropical ecosystem functioning and isolating possible effects of species diversity, due to multiple interactions among controlling state factors (i.e. climate, parent material, time and biota) that concurrently determine how many species coexist and how much C is sequestered by that plant species. This lack of knowledge limits our ability to predict tropical ecosystem functions and associated C sink-strength under future scenarios.

Ecological theory suggests that more diverse communities, such as in tropical forests should have the potential to exploit available resources more efficiently due to niche complementarity and positive species interactions^9^. Functional diversity should therefore increase through selection effects as communities containing a larger sample of the species pool should be more likely to contain highly productive species that contribute to ecosystem C storage^10^. Nonetheless, in the Amazon forest only a few but abundant species were reported to account for a disproportionate amount of the C sequestered by the ecosystem^11^, such that only 1% of the species found in the Amazon Basin accounted for half of the recorded tree stems^12^. Hence, how many species co-exist in a given area might be of secondary interest for ecosystem C storage, if these species are functionally equivalent in terms of C sequestration per area^13^. Accordingly, vegetation C storage in tropical forests might be rather independent of the total number of available plant species but was reported to be related to plant species composition via the presence or loss of keystone species in a given plant community^14^.

Plant community composition and the associated plant functional properties depend on the number of available niches shaped by local environmental factors^15^. Therefore, floristic communities with distinct functional properties may emerge on different parent materials and soil types in association with spatial variation of environmental factors, which act as filter on plant community composition due to differences in resource availability^16^. These filters systematically select for particular traits or trait syndromes^17^. For instance, nutrient-poor and stable environments generally select for species with a rather “stress-tolerant growth-strategy” whereas, more fertile and disturbed environments foster species with an “opportunistic growth-strategy”^18,19^. While, opportunistic and fast-growing species try to maintain high growth rates at the expense of costly structural tissues, stress-tolerant and slow-growing species produce relatively denser tissue and thus store relatively larger amounts of C per unit biomass^20^. As a result, environmental gradients in topography and disturbance regime may trigger differences in functional characteristics of the locally established plant community, such as the mean growth rate, lifespan, or wood density^21^ and thus determine the amount of C sequestered per area^22^.

Such feedbacks between belowground resource availability and aboveground vegetation dynamics^23^ have been shown to affect the turnover of C stored in AGB^24^ and thus should be considered when investigating relationships between biodiversity and biomass in tropical forests^4^. For instance, soil fertility generally promotes forest productivity, but the fast-growing species fostered at these sites tend to die younger^25,26^. How much C is stored per area therefore critically depends on plant community composition and associated life-history strategies of the local species pool^27,28^. In accordance, tropical aboveground C storage was found to vary with the assembly of species across landscape-scale gradients in geological substrate and topoedaphic factors affecting resource availability^29^. Such local-scale heterogeneity in biotic and abiotic factors might modulate the climate sensitivity of tropical forest stands in response to extreme climatic events^30^. Hence, accounting for local species composition and habitat heterogeneity among tropical forest stands should allow for a mechanistic representation of factors determining the spatial heterogeneity of tropical forest ecosystems and thus could be critical for projecting regional responses of tropical ecosystems to environmental change^31^. Our analysis is based on literature highlighting that by accounting for local-scale heterogeneity of tropical forest dynamics we should be able to drastically reduce uncertainty in large-scale estimates of tree species richness^32^ and forest C stocks^33^, as has been reported for modeling approaches (i.e. via disturbance regime^34^) and remote sensing products (i.e. via topographic position^35^).

**Figure 1:**
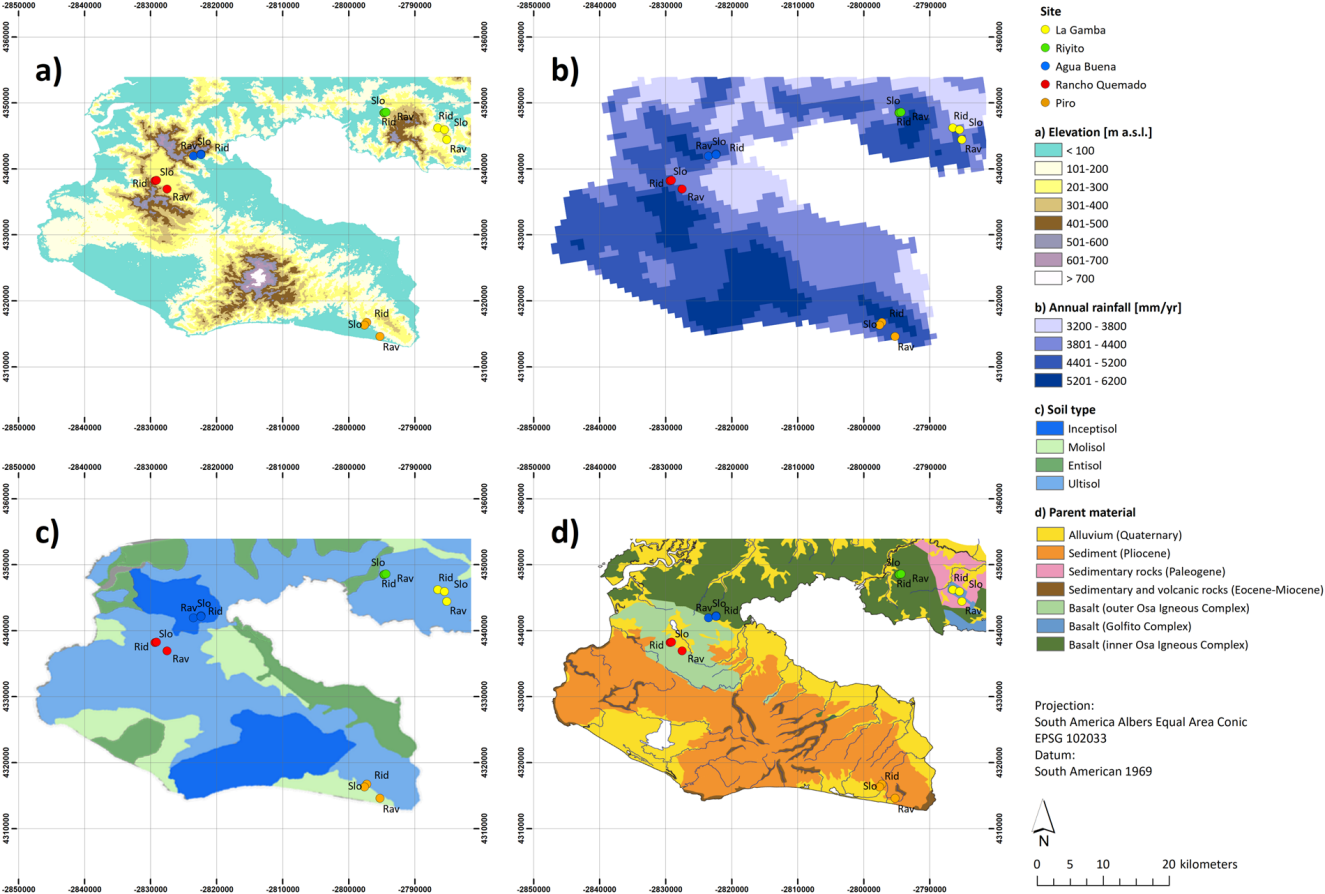
Environmental gradients and location of forest plots situated in the Área de Conservación Osa (ACOSA), southwestern Costa Rica (8°41′N, 83°13′W). Upper left panel (a): Elevation (m a.s.l.) based on SRTM ASTER data^58^. Upper right panel (b): Annual rainfall (mm yr^−1^) based on data from Climatologies at high resolution for the earth’s land surface areas (CHELSA; http://chelsa-climate.org)^56^. Lower left panel (c): Soil Type based on the map presented in Taylor *et al*.^29^. Lower right panel (d): Parent material based on an updated regional map first presented in Buchs *et al*.^79^. Point colors indicate respective location of forest plots spread across the study region. Geographic locations are depicted as colored symbols, i.e. La Gamba (yellow symbols), Riyito (green symbols), Agua Buena (blue symbols), Rancho Quemado (red symbols), and Piro (orange symbols). Forest habitat types are indicated by textual abbreviations, i.e. ridge forest plots (Rid), slope forest plots (Slo), and ravine forest plots (Rav) located in the Golfo Dulce region, southern Costa Rica. This map was created using QGIS Geographic Information System from Open Source Geospatial Foundation (URL http://qgis.org)^80^ and raster map data from the ASTER Global Digital Elevation Map (URL https://asterweb.jpl.nasa.gov/gdem.asp)^58^.

To that end, we surveyed fifteen undisturbed, lowland tropical forest sites across a geomorphologically heterogeneous region located at the Pacific slope of Costa Rica (Fig. 1). Based on this unique dataset comprising 7,752 individuals and 447 species of tropical trees, palms and lianas, we identified some of the confounding relationships between climatic and edaphic factors and how they affect tropical forest diversity and vegetation C storage of lowland tropical forests. We hypothesized that (i) landscape-scale heterogeneity of climatic and edaphic factors triggers differences in resource availability; such that (ii) local-scale gradients in resource availability affect the composition of plant species due to differences in their life-history strategy; and thus (iii) plot-scale vegetation C stocks differ in association with the locally dominant plant functional group (trees, palms, lianas) due to differences in their functional characteristics. Hence, we here explore how landscape-scale patterns of multiple and interrelated factors control tropical forest diversity and vegetation C storage, with the ultimate goal to increase our understanding of the underlying mechanistic factors determining tropical forest ecosystem functioning.

## Results

**Figure 2:**
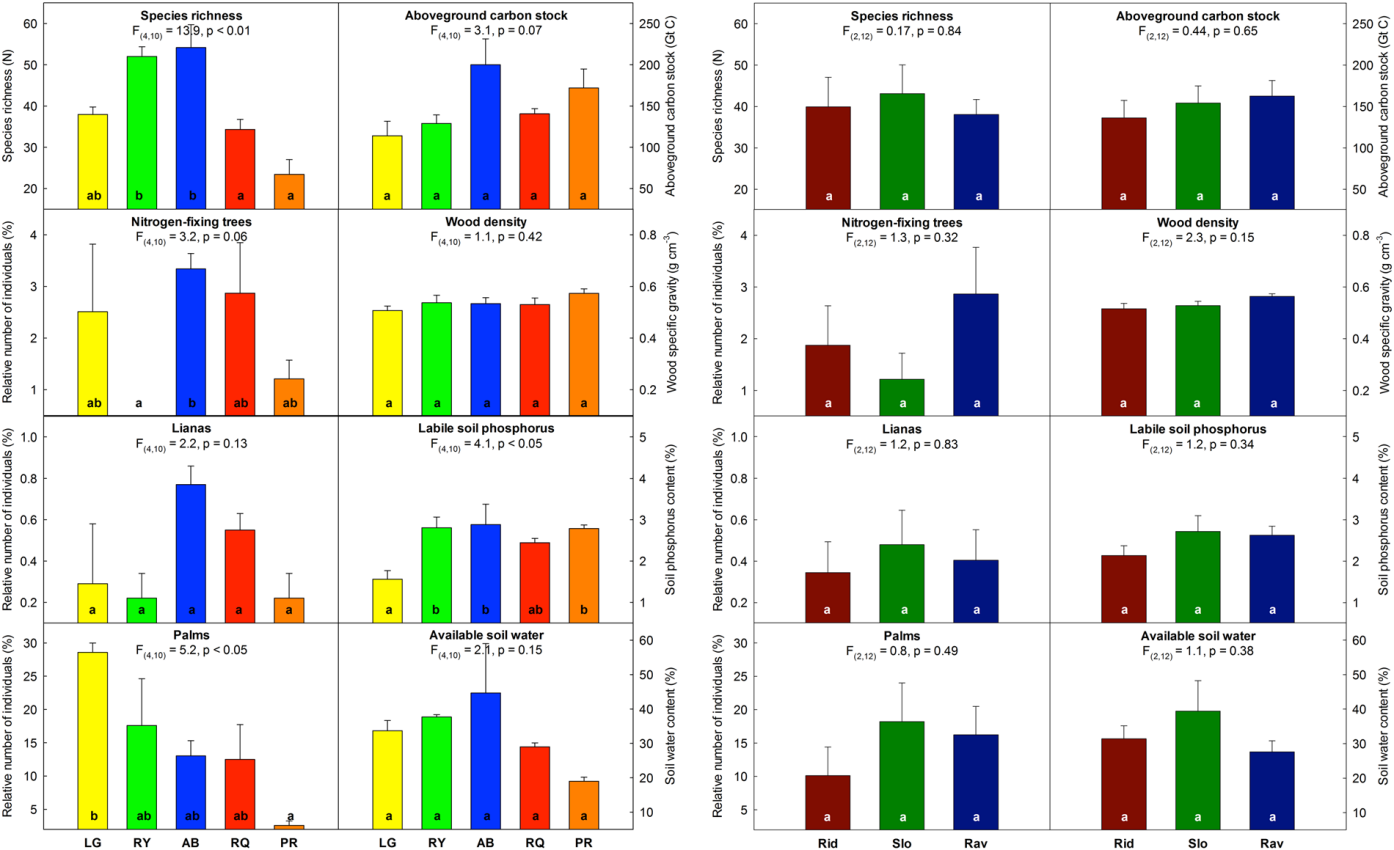
Tropical forest characteristics (per ha^−1^), i.e. plant species richness, aboveground C stock, community-weighted mean wood density, labile soil phosphorus, available soil water, as well as percentage of plant functional type, i.e. nitrogen-fixing tree species (Nfix), lianas (Liana) and palms (Palm) depicted for (i) each geographic location (left panel) i.e. La Gamba (yellow bars), Riyito (green bars), Agua Buena (blue bars), Rancho Quemado (red bars), and Piro (orange bars) and (ii) habitat type (right panel) i.e. ridge (Rid; darkred bars), slope (Slo; darkgreen bars), and ravine (Rav; darkblue bars). Statistically significant differences are indicated by respective letters (**a**–**c**) referring to Tukey’s HSD post-hoc test. For additional parameters please see Table S1 and for test statistics (i.e. F-ratio, degrees of freedom, p-values) please see Table S2.

**Figure 3:**
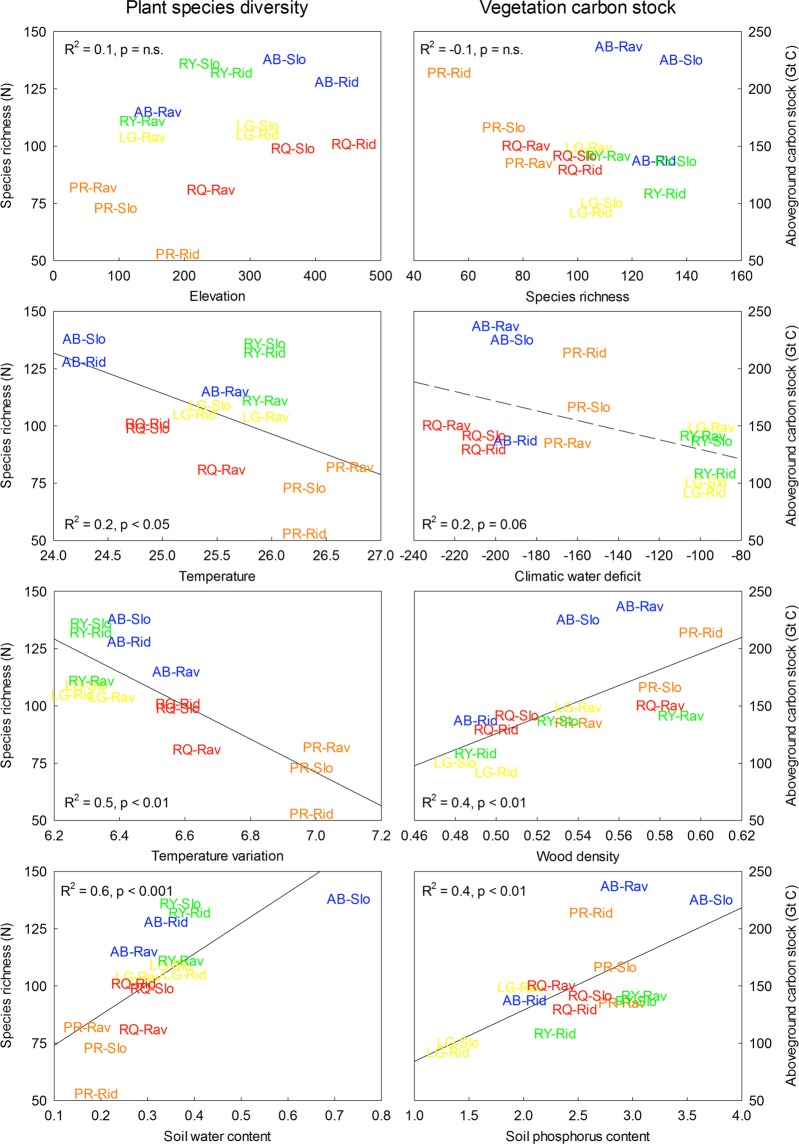
Effect of climatic and edaphic controls (i.e. elevation, mean annual temperature, mean annual temperature variation, climatic water deficit, soil water content, soil P availability and wood density) on tropical forest diversity (left panel) and vegetation C stock (right panel). Text label color refers to respective geographic location of forest sites located in southern Costa Rica, i.e. La Gamba (LG, yellow labels), Riyito (RY, green labels), Agua Buena (AB, blue labels), Rancho Quemado (RQ, red labels), and Piro (PR, orange labels). Text label ID refers to forest habitat type, i.e. ridge forest (Rid), slope forest (Slo), and ravine forest (Rav).

**Figure 4:**
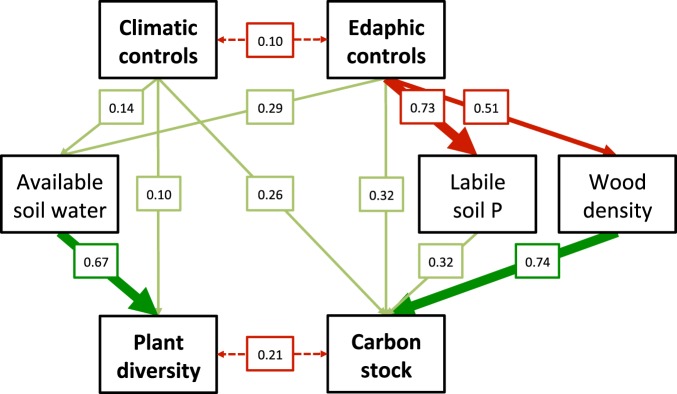
Structural equation model visualizing pathways among multiple controlling factors over tropical forest diversity and vegetation C storage (represented by the first two axes of principal components analyses, PC1: climatic controls, PC2: edaphic controls). Arrows indicate significant positive (green) or negative (red) relationships among variables. Arrow width indicates effect strength, and numbers are significant standardized path coefficients. The overall goodness of fit of the model was assessed by the difference between the model and the data, based on Fisher’s C statistic that follows a chi-square distribution (Fisher C = 17.47; df = 20; p = 0.622). Akaike’s information criterion (AIC) was used to compare alternative models and determine the most parsimonious model presented here (AIC = 67.47). The coefficient of determination R2 and intra-class correlation coefficient from generalized linear mixed-effects models can be indicated by pseudo-R2 values^81^. For mixed models, marginal R2 considers only the variance captured by the fixed effects (DIV = 0.52; ACD = 0.66), and conditional R2 by both the fixed and random effects combined (DIV = 0.94; ACD = 0.96).

Based on structural equation modeling we identified pathways among interrelated factors affecting tropical forest diversity and vegetation C storage across a geomorphologically and climatically heterogeneous landscape located in Costa Rica (Figs. 1 and S1). Our results highlighted differences in plant species richness and composition of plant functional groups (Figs. 2 and S2) associated with the variation of edaphic properties across the study region (Tables S1 and S2). While we did not find a positive relationship between tropical forest diversity and vegetation C storage (Fig. 3) our analyses revealed multiple and interrelated pathways among biotic and abiotic factors controlling the availability of water and nutrients along environmental gradients and thereby determining the biodiversity-ecosystem functioning relationship at the landscape-scale (Fig. 4).

Plant species richness (of trees, palms and lianas >10 cm in diameter) ranged from 69 to 127 species ha^−1^ (Table S1) and differed between geographic forest regions (Fig. 2). Plant species richness decreased with mean annual temperature (R^2^ = 0.27, P < 0.05) and more strongly so with increasing temperature variation (R^2^ = 0.64, P < 0.01). Plant species richness also significantly increased with increasing soil water content (R^2^ = 0.51, P < 0.001) from warmer and drier sites in the south to cooler and wetter sites in the north (Fig. 3). Results obtained from the path model indicated that plant species diversity was indirectly controlled by climatic factors via their effect on water availability and thus was directly and positively related to soil water content (Fig. 4).

Plant community composition differed among geographic forest regions (Fig. S2). Variance partitioning among factors explaining species dissimilarities between forest plots revealed a significant relation to climatic (4%), edaphic (17%) and spatial factors (13%) although residual variation was high (Fig. S3). As a result, the ecological dominance of plant functional groups differed among geographic forest regions (Table S2). The relative abundance of N-fixing tree species (F_(4,8)_ = 4.3, P < 0.05) and palms (F_(4,8)_ = 6.5, P < 0.01) was related to geographic forest region. The number of non-mycorrhizal (NM) tree species differed among topographic forest habitat type (F_(2,8)_ = 6.4, P < 0.05) and soil type (F_(2,2)_ = 32.3, P < 0.05). Habitat specialization of tree species associated with arbuscular-mycorrhizal fungi (AMF, F_(2,2)_ = 68.4, P < 0.01) and ecto-mycorrhizal fungi (EMF, F_(2,2)_ = 33.4, P < 0.05) varied with geologic substrate (Table S2). Nitrogen-fixing tree species and lianas were more abundant on basalt plots with relatively higher sand content and soil water availability, while palms were less abundant on sediment plots with relatively higher bulk density and lower soil water content, such that abundance of palms was inversely related to soil P availability (Fig. S2).

Aboveground vegetation C storage ranged from 114 to 172 t ha^−1^ in association with climatic and edaphic factors and differences in plant community composition across the study region (Fig. S4). Vegetation C storage increased with community-weighted mean wood density (R^2^ = 0.43, P < 0.01) and soil phosphorus content (R^2^ = 0.45, P < 0.01), but tended to decrease with increasing climatic water deficit (R^2^ = 0.24, P = 0.06) across the study region (Fig. 3). Results obtained from the path model suggest that aboveground vegetation C storage was indirectly controlled by edaphic properties via their effect on nutrient availability and plant community composition (Fig. 4).

## Discussion

We found that tropical forest diversity and vegetation C storage was strongly related to landscape-scale patterns in climate and edaphic properties across a geomorphologic heterogeneous region located in southern Costa Rica (Fig. 1). Our findings corroborate previous studies of edaphic gradients across Amazonia^15^, Borneo^35^, south-eastern Ecuador^36^ and south-western Costa Rica^29^, which indicated that vegetation characteristics varied in association with the regional variation of soil parent material, landform and soil types across the study region. Based on these findings we hypothesized that tropical forest diversity should be related to the abiotic equivalent of biological diversity, i.e. so-called geodiversity^37^. While similar relationships have been reported for a number of tropical forest sites^15,29,35,36^, our analysis based on statistical path modeling revealed how interrelated pathways among multiple controlling factors shape tropical forest diversity and determine the amount of C stored in vegetation biomass. Our findings highlight that the underlying controlling factors shaping plant community composition and thus affecting the amount of C sequestered by the ecosystem should be considered when attempting to predict tropical forest ecosystem functioning in a future climate. Hence, our results could be used to improve currently applied vegetation models by accounting for factors determining the spatial heterogeneity of tropical ecosystem processes and thus should allow to refine projections of tropical forest ecosystem functioning under future climate change scenarios.

Our analysis based on statistical path modeling revealed that beside climatic factors, such as temperature and precipitation, edaphic parameters, such as soil texture and chemistry, emerged as important controls over tropical plant community composition. Such relationships between soil edaphic properties and plant species composition have been proposed for other tropical regions and can be explained by the fact that local edaphic properties affect the resource availability of water and nutrients in tropical soils and thereby select for plant communities with distinct ecological functions on different parent materials and soil types^16^. We here found pronounced differences in the relative abundance of plant functional groups (i.e. trees, palms and lianas) and mycorrhizal association (AMF and EMF) of tropical plant communities associated with geomorphological heterogeneity of the study region. Our findings for lowland tropical forests confirm studies conducted in pre-montane tropical forests, which reported differences in the taxonomic and functional composition of tropical tree communities in association with gradients in rainfall seasonality and soil resource availability^38^. Accordingly, we found that palms were more abundant on soils with high bulk density and low soil phosphorus availability, while N-fixing tree species were found on relatively less dense soils with high soil water availability (Fig. 2). Hence, our findings support the hypothesis that differences in the weathering of bedrock material triggered differences in resource availability and thus lead to differences in plant functional community composition across the landscape.

Landscape-scale variation in functional community composition results from local-scale specialization of a given species in response to its climate envelope and the filtering of the locally available species pool by physical and chemical soil properties^39^. We here found differences in plant functional community composition across topoedaphic gradients and more generally that sites with lower resource availability contained less diverse plant communities than those with ample soil water and nutrient supply (Fig. 3). Hence, our findings point to the proposed positive relationship between resource availability and plant species richness via soil resource-based niche differentiation^15,23^. For instance, studies conducted in Africa^40^ and Guyana^41^ found that belowground soil properties accounted for the variability in aboveground forest structure because differences in resource availability and nutrient retention determined forest dynamics due to different life-history strategy of tree species adapted to different positions along environmental gradients^42^. Such shifts in taxonomic and functional species composition along topoedaphic gradients^35,36^ likely reflect trade-offs among investment strategies along the plant economics spectrum^18,43^. With increasing resource limitation tropical tree communities become dominated by species with conservative resource-use strategies geared towards maximum survival as opposed to rapid biomass gain^44^. Hence, one might expect a slow, survival-oriented strategy with higher wood density in nutrient poor ridge forests. However, we here found the opposite pattern of increasing wood density from ridge to ravine forests. Our counterintuitive finding can be explained by the observation that across topoedaphic gradients, ridge forests are generally characterized by strongly acidic and highly weathered Ultisols, as well as a relatively higher number of individuals within smaller size-classes. The latter would indicate high disturbance rates and an increased turnover of individual stems (e.g. due to drought or storms) and thus lead to development of forest gaps at more exposed ridge forest sites^45^. In such stressful environments, individuals would invest in rapid height growth at the cost of producing structural tissue, which would explain the observed lower wood density at ridge forest sites. Indeed, a foregoing study conducted in the same region reported differences in turnover rates and tree mortality among topographic forest habitat types, such that forest plots situated uphill exhibited a higher turnover of tree stems compared to downslope forest sites^30^. Our observation of differences in community-weighted mean wood density from downslope ravine to uphill ridge forests therefore likely indicates that local edaphic gradients select for species with different life-history strategy and thereby shape plant community composition and associated vegetation C storage along environmental gradients in resource availability.

Preceeding research evaluating the effects of biodiversity on ecosystem C storage reported that plant species composition was a critical determinant of vegetation C storage in tropical forests. For instance, a study assessing the impact of tree species loss on tropical C storage by simulating potential extinction scenarios found huge variation in aboveground C stocks depending on which species where lost in the extinction scenario^14^. These results indicate that tropical C storage will strongly depend on future species composition and furthermore confirms the linkage of vegetation characteristics and ecosystem functioning across topo-edaphic gradients in resource availability^23^. In line with these findings it has been proposed that endogenous disturbance levels, species composition, and forest productivity determine a self-maintaining forest dynamic feedback mechanism initiated by edaphic conditions across the Amazon Basin^19^. Accordingly, it has been proposed that ecosystem heterogeneity in association with spatial variation in soil texture explained observed patterns of variation in forest biomass, composition, and dynamics and thus determined the ecological resilience of forest ecosystems to climate change^46^. Indeed, a study conducted in the same region reported differential sensitivity of tropical lowland forest stands to an El Niño short-term drought event in association with local topography (water availability) and disturbance regime (species composition) and thus concluded that local site characteristics will likely prevent uniform responses of tropical lowland forests to projected global changes^30^. These findings once again indicate that it is crucial to account for local habitat heterogeneity when attempting to predict tropical ecosystem functioning under future scenarios because the functional response of tropical tree communities depends on both large-scale climatic signals as well as local edaphic properties affecting the availability of water and nutrients (Fig. 4).

So far, traditional large-scale projections of global change effects on tropical forests typically ignore the spatial heterogeneity of edaphic parameters and temporal changes in plant community composition. As a consequence, most of the currently applied approaches fail to accurately represent crucial ecosystem processes, such as vegetation C storage, because remote sensing techniques typically integrate over large spatial areas thus averaging out local landscape heterogeneity, and vegetation models usually ignore heterogeneous functional responses of plant communities to climatic factors. Addressing these challenges, recent crosscutting approaches linking remote sensing, forest modeling and field inventories demonstrated potential avenues for implementing spatial habitat heterogeneity and functional diversity in model based projections^34^ and remote sensing approaches^35^. For instance, implementation of functional trait diversity in dynamic vegetation models has recently been accomplished^47^ thereby providing a toolbox to improve our understanding of tropical forest dynamics and to generate more reliable long-term projections of its response to climate change. As a result, such novel approaches should yield more realistic scenarios of tropical ecosystem functioning in a future climate and help to improve current biodiversity conservation and management strategies for tropical forest ecosystems.

Eventually, our findings point to some of the remaining challenges for developing improved dynamic vegetation models. For instance, our results indicated strong interactions between environmental variables, functional traits of individuals, and taxonomic and functional community composition in tropical forests. A particularly challenging aspect is that plant functional traits may respond relatively quickly to climate change via acclimation of individuals, whereas shifts in community composition will show more pronounced temporal lags^48^, and it is not obvious how these processes might interact^49^. Overall, we found considerable unexplained variation in taxonomic community composition between tropical lowland forest stands, which further highlights the importance of accounting for additional factors, such as competition among coexisting individuals. To resolve these issues, more empirical research is needed on the plasticity of plant functional traits within individuals and the genetically determined variability in the community, as well as on the demography and disturbance processes driving community dynamics in tropical forests.

## Methods

### Study area and selection of permanent inventory plots

The study was conducted in tropical lowland forests located between 50 and 450 m a.s.l. in the Área de Conservación Osa (ACOSA) at the Pacific slope of southwestern Costa Rica (08.6°N, 83.2°W). The study region represents the largest remaining, intact Pacific lowland tropical forest in Central America^50^ and is considered a biodiversity hot spot with 700 tree species among 2369 species of vascular plants recorded in total^51^. The area also harbours a high diversity of fauna with 140 mammal species and 205 bird species, including important seed dispersers, such as the Central American Tapir (*Tapirus bairdii*), peccaries, deers, procyonids and monkeys^52^. Records of human settlements and colonization date back to 1000 years BC, but the historical growth of the human population has been slow due to the remoteness to the administrative center of the country. Part of the forest was cleared for cattle ranching on pastures and plantations with rice, banana, oil palm and timber during the 19^th^ century. While there is a history of logging, gold mining and hunting in the region^53^, the forest plots investigated are clearly old-growth forests and show no evidence of past human disturbance in the more recent past.

The terrain is characterized by parent material originating from the Cretaceous, Tertiary and Quaternary (basalt, alluvium and sediment, respectively) and is divided into six different landforms (denudational, volcanic, alluvial, structural, littoral, tectonic) and four soil orders (Entisols, Inceptisols, Mollisols and Ultisols)^54^. The dominating, highly weathered, strongly acidic Ultisols on ridges and upper slopes are replaced by younger, moderately weathered Inceptisols in ravines and lower slopes and little developed Mollisols in fluvial deposits^54^. Daily climatologic data for temperature and precipitation (starting 1997) are available from La Gamba field station (https://www.lagamba.at/en/tropical-field-station/scientific-data-of-the-golfo-dulce-region). For the period 1998–2017 mean annual temperature (MAT) ranged from 24.3 to 26.7 °C and mean annual precipitation (MAP) varied between 3959 and 5007 mm per year, with no month receiving less than 180 mm on average. However, across the study region precipitation seasonality varies and rainfall decreases from the mainland towards the southern end of the Osa peninsula^55^. We therefore obtained data of mean annual temperature (MAT), temperature seasonality (MATvar), mean annual precipitation (MAP) and precipitation seasonality (MAPvar) from CHELSA database (http://chelsa-climate.org)^56^ at 2.5 arc-minutes (~5 km) spatial resolution. In addition, the long-term climatic water deficit (CWD), i.e. the difference between rainfall and evapotranspiration during dry months, was extracted from a global climate layer for the long-term average of CWD at 2.5 arc-minute resolutions (http://chave.ups-tlse.fr/pantropical_allometry.htm)^57^. Furthermore, to assess the spatial heterogeneity and complex geomorphology of parent material and prevailing soil types across the region we extracted elevation (ELE), slope (SLO), and aspect (ASP) from the ASTER Global Digital Elevation Map v 2, (https://asterweb.jpl.nasa.gov/gdem.asp)^58^.

The selection of permanent inventory plots was based on a stratified sampling design^59^, i.e. the set of ecosystem types to be studied was selected upon a thorough evaluation of the relative spatial contribution of different ecosystem types in the study region. The dominant regional ecosystem types were classified as broad-leaved evergreen well-drained lowland forests (38.3%), dense tropical evergreen well-drained lowland (woody) herbland dominated by graminoids (pastures) (22.0%) and dense tropical broad-leaved evergreen well-drained lowland shrubland with early to late successional regrowth (9.7%)^50^. A classification of regional forest types based on remote sensing found that dominant forest ecosystem types were old-growth forest stands located in ridge (3.2%), slope (84%) and valley (2.8%) position as well as late successional regrowth forest stands (9.7%) near populated agricultural areas in the region^52^. Hence, in order to account for the spatial heterogeneity of the study area permanent inventory plots were established in undisturbed forest stands differing in topography, i.e. ridge (rid), slope (slo) and ravine (rav) positions, which were replicated across five geographic locations (La Gamba, Riyito, Agua Buena, Rancho Quemado and Piro). Forest plots were of 1-ha size and subdivided into subplots (10 × 10 m) following methodological standards^60^. In some cases, such as for ravine forest plots situated along small streams, plot shape was adapted to the physiography of the terrain, and therefore ranged from regular (100 × 100 m) to irregular shapes. In the following, we analyze effects of climatic and edaphic drivers on plant species diversity and C stored in aboveground biomass based on data obtained from 1-ha forest inventory plots.

### Species composition and forest inventory data

A total of 11,786 individuals were recorded in the 2012–2015 census of which 85% were identified to the species and 96% to the genus level, thus representing 485 species, 280 genera, and 77 families of trees, palms and lianas, of which 7,752 individuals and 447 species were located in undisturbed old-growth forest plots analyzed in this study. Data collection involved all living stems with a diameter at breast height (i.e. 1.3 m DBH) ≥ 10 cm. For trees with buttresses or other deformations, the point of measurement (POM) was raised above the end of the buttress or deformation, following standardized protocols^61,62^. Tree diameter was measured with a diameter tape and tree height was calculated via triangulation, measuring the distance to the tree stem and the angle to treetop with an ultrasound instrument (Vertex IV, Haglöf). Wood density was compiled from a global database of tropical forest wood density^63^, using genus or family level data when species identity or species-level wood-density data was not available. Plot (= community) means of wood density was calculated based on weighing by tree basal area. Aboveground biomass (AGB) was calculated for each 1-ha plot using the R code provided in the BIOMASS package^64^ based on the moist forest allometric equation^57^ (AGB = 0,0509 × *ρ* D2 H), where AGB is the biomass of each stem (kg), *D* is stem diameter (cm), *ρ* is stem wood density (g cm^−3^) and *H* is stem height (m). To convert AGB into C, we assumed that dry biomass is 47.3% carbon, based on recent studies conducted in lowland forests in Panama^65^. The data have been curated and shared via the ForestPlots.net database^66^. Plant samples collected for taxonomic identification were deposited at the Herbarium of the Biology School of the University of Costa Rica (USJ herbarium code). Functional species composition (i.e. the ecological dominance of different plant functional groups between the investigated forest regions and habitat types) was evaluated by classifying species based on symbiotic interactions (mycorrhiza, N-fixing symbionts) that may enhance their competitive advantage in nutrient uptake^16^. According to the proposed classification scheme we categorized species as ectomycorrhizal (EM) or arbuscular mycorrhizal (AM) if they occurred in families known to contain EM or AM taxa^67–69^. The remaining species were assigned N-fixing taxa (Nfix) if belonging to the legume sub-families Mimosoideae and Papilionoideae or else as non-mycorrhizal (NM) taxa^16^.

### Soil sampling and chemical analysis

In each of the twenty 1-ha inventory plots, we sampled five individual soil cores (2.5 cm diameter, 0–15 cm below the litter layer) along five transects interspaced by ~10 m, manually picked coarse and fine roots to estimate belowground root biomass and pooled sieved soils to a compound sample. Soil samples were collected in the mid-wet season (July 2012). Volumetric soil water content (VWC) and bulk density (BD) were determined by drying a known volume of soil at 105 °C^70^. Soil water content (SWC), electrical conductivity (EC) and soil temperature was assessed *in-situ* using a WET Sensor type WET-2 connected to an HH2 Moisture Meter (Delta-T Devices Ltd). Soil extracts from each 1-ha plot were analyzed for pH, total C, N and P, extractable inorganic N (NH4 and NO3) and P, extractable cations (Al, Ca, Fe, K, Mg, Mn and Na), effective cation exchange capacity (ECEC, i.e. the sum of extractable cations), and total exchangeable bases (TEB, i.e. the sum of Ca, K, Mg and Na). Base saturation was calculated as (TEB/ECEC) x 100. Soil pH was measured in water with an ISFET electrode in a 1:2 dry soil:water ratio (Sentron ArgusX pH meter). Soil cations were determined by extraction with 0.1 M BaCl_2_. Total soil carbon (soil C) and nitrogen content (soil N) were determined by elemental analysis (Thermo EA 1110, CE Instruments). Total soil phosphorus content (total soil P) and cations (Al, Ca, Fe, K, Mg, Mn and Na) were analyzed after acid digestion with nitric acid and perchloric acid by inductively coupled plasma optical emission spectrometry (ICP-OES Optima 5300DV). Labile inorganic nitrogen (ammonium, nitrate) and labile inorganic phosphorus (phosphate) was determined by extracting fresh soils with 0.5 M K_2_SO_4_. The concentration of ammonium and nitrate was determined colorimetrically; ammonium was determined based on the Berthelot reaction; nitrate was determined by reduction with acidic VCl_3_ and the Griess reaction. Total labile (organic and inorganic) soil phosphorus (labile soil P) concentration was determined by oxidation of extracts with persulfate, and subsequent quantification with the malachite green method on a microplate reader^71^.

### Statistical analysis

We investigated edaphic (parent material, topography, soil texture and soil chemistry) and climatic (mean annual temperature, temperature seasonality, mean annual precipitation, precipitation seasonality, climatologic water deficit) controls on tropical forest species composition and associated aboveground vegetation C storage. We used linear mixed effect models (accounting for fixed and random effects due to the nested study design of replicated forest types within each forest region) to test for variation of forest structure (i.e. stem number, mean diameter above breast height, total basal area, max. tree height, aboveground biomass, root biomass, leaf area index, community-weighted mean wood density, aboveground C stock) and species composition (i.e. species number, species diversity, species composition) among categorical variables such as “forest region”, “forest type”, “parent material”, “soil type”, while accounting for nested random effects of forest habitat types (i.e. situated in ridge, slope, ravine position) within respective forest region (i.e. located in La Gamba, Riyito, Agua Buena, Rancho Quemado, Piro). We further used multiple linear regression models investigating the response of tropical forest diversity and vegetation C storage to the above factors with respective fixed effects “climate”, “parent material”, and “soil type” while accounting for random effects “geographic location” and “habitat type”. Assessing linear model assumptions we tested for skewness, kurtosis and heteroscedasticity^72^. We applied principal component analysis (PCA) to reduce collinearity among related factors such as edaphic and climatic variables across environmental gradients and thus computed respective principal components of climate (PC1) and soil variables (PC2). PC1 accounted for 32.9% of variation among the variables MAT, MAP and CWD. PC2 captured 21.8% of variation in ELE, BD, ECEC and relative soil clay/sand/silt content.

We used structural equation modeling (SEM) to investigate interrelated parameters and dissecting direct from indirect controls over plant species richness and C stored in AGB. This multivariate statistical tool allows identifying relationships among interrelated factors based on the covariance among variables to build and test models^73^. We applied a fully documented open-source package for piecewise SEM (pSEM), a practical implementation of confirmatory path analysis within the R programming language, which extends this method to all current (generalized) linear, (phylogenetic) least-square, and mixed effects models (accounting for fixed and random effects), relying on familiar R syntax^74^. Model optimization was an iterative process analogous to stepwise multiple regression and Akaike’s information criterion (AIC) was used to compare alternative models and determine the most parsimonious model^75^. We removed insignificant pathways from the base model, which included all significant parameters identified by the linear regression models, in a stepwise manner, aiming at improvement of the overall model fit^76^. The overall goodness of fit of the model was tested based on comparison of the Fisher’s C statistic to a chi-square distribution (Fisher C = 17.47; df = 20; p = 0.622) thus representing little difference between the model and the data. All statistical analyses were implemented using the R statistical software^77^ and respective packages “Biomass”, “BiodiversityR”, “nlme”, “piecewiseSEM”, “vegan”. For additional information on statistical analysis and methodological details please see the supplementary material available in the online version of this paper.

## Supplementary information

Supplementary Material.

## Data Availability

The datasets generated during and/or analyzed during the current study are available in the Forest Observation System (FOS) repository^78^, http://forest-observation-system.net.
