# Supplementary material for: Climatic and edaphic controls over tropical forest diversity and vegetation carbon storage
Source: Sci Rep. 2020 Mar 19;10:5066. doi: 10.1038/s41598-020-61868-5 (PMC7081197; doi:10.1038/s41598-020-61868-5)
Supplement: Supplementary file 1 — Supplementary Material. [file 41598_2020_61868_MOESM1_ESM.docx]

SREP-18-30060C Supplementary Material

**Title**

Climatic and edaphic controls over tropical forest diversity and vegetation carbon storage

**Author list**

Florian Hofhansl*^1^, Eduardo Chacón-Madrigal^2^, Lucia Fuchslueger^3^, Daniel Jenking^4^, Albert Morera^5^, Christoph Plutzar^6,7^, Fernando Silla^8^, Kelly M Andersen^9^, David Buchs^10^, Stefan Dullinger^6^, Konrad Fiedler^6^, Oskar Franklin^1^, Peter Hietz^11^, Werner Huber^6^, Carlos A. Quesada^12^, Anja Rammig^13^, Franziska Schrodt^14^, Andrea Vincent^2^, Anton Weissenhofer^6^, Wolfgang Wanek^15^

**Affiliations** *Corresponding author [hofhansl@iiasa.ac.at](mailto:hofhansl@iiasa.ac.at)

^1^International Institute for Applied Systems Analysis (IIASA); Schlossplatz 1, A-2361 Laxenburg, Austria; ^2^Escuela de Biología, Universidad de Costa Rica, San José, Costa Rica; ^3^Department of Biology, Plants and Ecosystems, University of Antwerp, Antwerp, Belgium; ^4^Escuela de Agronomía, Universidad de Costa Rica, San José Costa Rica; ^5^Laboratory of Applied Tropical Ecology, National University of Costa Rica, Costa Rica; ^6^Department of Botany & Biodiversity Research, University of Vienna, Austria; ^7^Institute of Social Ecology, University of Natural Resources and Life Sciences, Vienna, Austria; ^8^Area of Ecology, Faculty of Biology, University of Salamanca, Spain; ^9^Nanyang Technological University, Asian School of the Environment, 50 Nanyang Avenue, 639798, Singapore; ^10^School of Earth and Ocean Sciences, Cardiff University, Park Place, Cardiff, CF10 3AT, UK; ^11^Department of Integrative Biology and Biodiversity Research, Institute of Botany, University of Natural Resources and Life Sciences, Vienna, Austria; ^12^Instituto Nacional de Pesquisas da Amazônia, Coordenação de Dinâmica Ambiental, Avenida Ephigenio Salles 2239, Aleixo - 69000000, Manaus, AM; Brasil; ^13^Technical University of Munich, TUM School of Life Sciences Weihenstephan, Hans-Carl-v.-Carlowitz-Platz 2, 85354 Freising, Germany; ^14^School of Geography, University of Nottingham, University Park, Nottingham, NG7 2RD; ^15^Department of Microbiology & Ecosystem Science, Division of Terrestrial Ecosystem Research, University of Vienna, Austria.

**Supplementary Results**

In this study we investigated the impact of local environmental controls, such as climate, parent material, topography and soil types on plant species richness (alpha diversity), turnover among forest sites (beta diversity), species composition of plant functional groups (functional diversity), and carbon stored in aboveground biomass (i.e. vegetation C stock) between permanent 1-ha inventory plots located in the Golfo Dulce region, southern Costa Rica (Fig. 1, Fig. S1). We found that species diversity, plant community composition and functional composition, i.e. trees, palms, lianas, nitogen-fixing plants and myccorhizal association, as well as soil water content and phosphorus availability varied among geographic regions and forest habitat types (Fig. 2, Fig. S2) and used statistical analysis to dissect interrelated relationships among environmental controlling factors (Fig. 3, Fig. S3) over plant species diversity and vegetation C stocks in topical lowland forests (Fig. 4, Fig. S4).

Environmental factors such as climate, parent material, topography and soil type potentially controlling plant species diversity and vegetation C stock varied among permanent inventory plots established in the Golfo Dulce region located in southwestern Costa Rica (Table S1). Mean annual temperature ranged from 24.7 to 26.4 °C, mean annual precipitation from 4262 mm to 4891 mm and climatic water deficit varied between -95.4 and -211.9 mm yr-1. Most climatic factors including mean annual temperature (F(_4,8_)=18.9, P<0.001), mean annual precipitation (F(_4,8_)=6.7, P<0.01) and climatic water deficit (F(_4,8_)=431, P<0.001) differed among forest regions, while edaphic properties, such as sand content (F(_4,8_)=8.0, P<0.01), clay content (F(_4,8_)=4.8, P<0.05), and effective cation exchange capacity (F(_4,8_)=42.7, P<0.05) varied between forest types (Table S2).

We further report significant differences in plant species richness (F(_4,8_)=15.2, P<0.001), plant species diversity (F(_4,8_)=13.6, P<0.001), stem diameter (F(_4,8_)=4.8, P<0.05) and AGB (F(_4,8_)=3.9, P<0.05) among forest regions (Table S2). We moreover found differences in the abundance of dominant tropical plant functional groups, i.e. trees, palms and lianas >10 cm in diameter at breast height with >100 individuals per 1 ha plot (Table S3). Our results indicate differences in taxonomic species composition, functional community composition, and aboveground vegetation C stocks among respective 1 ha inventory plots established across the Golfo Dulce region, southern Costa Rica (Fig. S1).

Differences in tree species composition among forest plots were assessed using hierarchical cluster analysis and non-metric multidimensional scaling (NMDS) based on a Bray–Curtis “*dissimilarity matrix”*, using square root-transformed species abundance data to visualize compositional variation among forest regions and habitat types (Fig. S2). Ordination of NMDS displayed that hierarchical clusters were related to geographic location and topographic habitat type (Fig. S2). The intermediary plots (i.e. LG, RY, AB) formed a distinct cluster from the northern plots (RQ) and the southern plots (PR) on the other side. Sediment plots were characterized by distinct community composition with low species diversity and overall abundance of palms (Fig. S2; Table S3). The southernmost latitude and low elevation site (PR) was characterized by relatively lower soil water content and high variation in mean annual temperature, whereas the northern latitude sites with higher water availability (AB, RQ) showed a distinct community composition with higher abundance of nitrogen fixing tree species and lianas, and the site with lowest phosphorus availability (LG) showed highest abundance of palms (Fig. 2, Table S4).

We used variance partitioning (Fig. S3) of respective first PCA axis (Fig. S4) of climatic variables, i.e. temperature, precipitation (PCA_climate_); edaphic properties i.e. soil texture, soil chemistry (PCA_soil_); geology i.e. parent material, soil type (PCA_geo_); and spatial distance, i.e. geographic distance (spatial PCNM) to explore respective contributions of factors explaining species turnover among respective 1ha plots. We found that spatial distance, geological factors, edaphic properties and climatic variables explained up to one third of the variation in species composition among forest plots. While spatial factors covered 13% of total variation, environmental variables i.e. parent material, soil type and climate had lower explanatory power (11%, 6% and 4% respectively). Although, the explanatory power of the sum of environmental factors surmounted those of spatial distance, the residual variation was high (75%), therefore indicating the influence of non-environmental or unidentified environmental drivers on species composition (Fig. S3). Nonetheless, the first two axes of a principal component analysis (PCA) of the investigated environmental variables explained 50% of the variance in the data set (PC 1 0.32; PC2 0.19; PC3 0.13; PC4 0.12; PC5 0.10), and grouped forest plots according to differences in topoedaphic properties (PC 1) and climatic variables (PC 2). Although there was no clear pattern, plots from higher elevations which was associated with relatively higher clay content were located opposite to plots from lower regions in association with higher sand content and nutrient availability (Fig. S4).

**Supplementary Figure legends**

**Figure S1.** Location of forest plots established across the Golfo Dulce region, southern Costa Rica (8°41´N, 83° 13´ W).

**Figure S2.** Ordination based on nonmetric multidimensional scaling (NMDS) showing variation in community composition of recorded tree species (points) among geographic locations (upper panel) i.e. La Gamba (yellow polygon), Riyito (green polygon), Agua Buena (blue polygon), Rancho Quemado (red polygon), Piro (orange polygon) and forest habitat type (lower panel), i.e. ridge forest plots (darkred polygon), slope forest plots (darkgreen polygon), ravine forest plots (darkblue polygon) and secondary forest plots (darkgrey polygon) located in the Golfo Dulce region southern Costa Rica.

**Figure S3.** Venn diagram showing variance partitioning among factors explaining species dissimilarities among forest plots by accounting for respective principal components of climatic variables (i.e. temperature, precipitation), edaphic properties (i.e. soil texture, soil chemistry), geology (i.e. parent material, soil type) and spatial distance (geographic distance). Note that residual variation was high indicating the influence of other factors on species composition.

**Figure S4.** Principal component analysis (PCA) showing variation of environmental factors, such as climatic variables i.e. mean annual temperature (MAT), mean annual precipitation (MAP), climatic water deficit (CWD), as well as, topoedaphic parameters i.e. elevation (ELE), sand content (sand), silt content (silt), clay content (clay), soil bulk density (BD) and effective cation exchange capacity (ECEC) among forest plots. Direction and length of vectors indicates relative effect size of respective environmental controlling factor. Point color refers to geologic substrate (i.e. parent material). Text label color refers to respective geographic location i.e. La Gamba (yellow labels), Riyito (green labels), Agua Buena (blue labels), Rancho Quemado (red labels), and Piro (orange labels) of forest stands located in the Golfo Dulce region, southern Costa Rica.

**Supplementary Table legends**

**Table S1.** Summary statistics (i.e. mean ± standard error) for parameters evaluated in this study based on 1-ha forest inventory plots established in the Área de Conservación Osa (ACOSA) at the Pacific slope of southwestern Costa Rica (cf. Figure 1). Details are given for each forest region i.e. Agua Buena (AB), La Gamba (LG), Piro (PR), Rancho Quemado (RQ), Riyito (RY) and forest habitat types i.e. ridge (Rid), slope (Slo), ravine (Rav). Respective means and standard errors are presented for each forest region and habitat type, categorized according to location (Latitude, Longitude, Elevation), climatic variables (mean annual temperature (MAT), temperature variability (MATvar), mean annual precipitation (MAP), rainfall variability (MAPvar)), and edaphic properties, (volumentric water content, sand, silt and clay content, soil pH, cation echange capacity (ECEC), soil carbon, soil nitrogen, soil phosphorus content). Vegetation characteristics of trees, palms and lianas with stems >10 cm identified to species level and vegetation structure (number of stems, basal area, diameter at breast height, maximum average tree height, leaf area index, wood density, wood biomass, root biomass, aboveground vegetation carbon stock) and species composition (species number, Fisher’s α diversity), as well as, relative abundance of each functional group, such as ectomycorrhizal (EM), arbuscular mycorrhizal (AM), non-mycorrhizal (NM) and N-fixing taxa (Nfix), lianas (Liana) and palms (Palm) was quantified.

**Table S2.** Results of linear mixed effects models accounting for nested factors, such as forest habitat types (i.e. ridge, slope, ravine) within respective forest region (i.e. Agua Buena, La Gamba, Piro, Rancho Quemado, Riyito), and categorical level “Forest region”, “Forest type”, “Geological substrate”, “Soil type”. Significant differences are indicated by respective F-ratio (numDF, denDF), p-value and level of significance indicated by asterisk (p<0.05 *; p<0.01 **; p<0.001 ***). The Bonferroni correction method was applied to avoid Type I-error inflation.

**Table S3.** Abundance of dominant plant species (with > 100 individuals per 1 ha plot) indicating differences in taxonomic species composition (Table S1) and functional community composition (Table S2) among forest regions, i.e. Agua Buena (AB), La Gamba (LG), Piro (PR), Rancho Quemado (RQ), Riyito (RY) and habitat types, i.e. ridge (Rid), slope (Slo), ravine (Rav) located in the Golfo Dulce region, southern Costa Rica.

**Table S4.** Number of individuals in respective functional group with distinct mycorrhizal association, i.e. arbuscular (AMF), ectomycorrhizal (EMF), non- mycorrhizal (NM) or plant life form, i.e. tree, palm, liana and nitrogen fixing species (Nfix) according to classification presented in Prada et al., (2017). Plot ID refers to 1-ha inventory plots and respective forest region i.e. Agua Buena (AB), La Gamba (LG), Piro (PR), Rancho Quemado (RQ), Riyito (RY) and habitat type i.e. ridge (Rid), slope (Slo), ravine (Rav) located in the Golfo Dulce region, southern Costa Rica.

**Supplementary Figure S1**

**
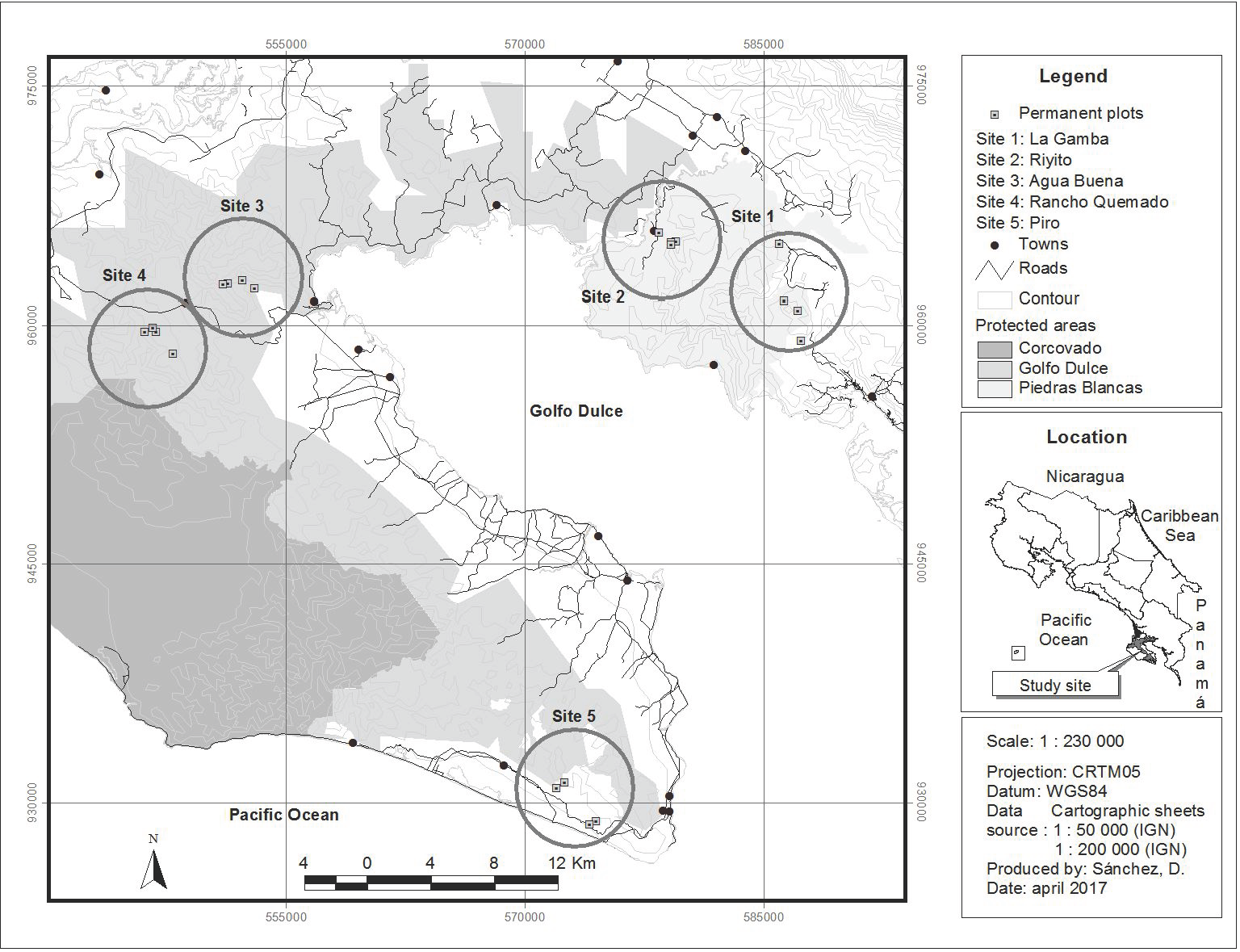
**

**Supplementary Figure S2**

**
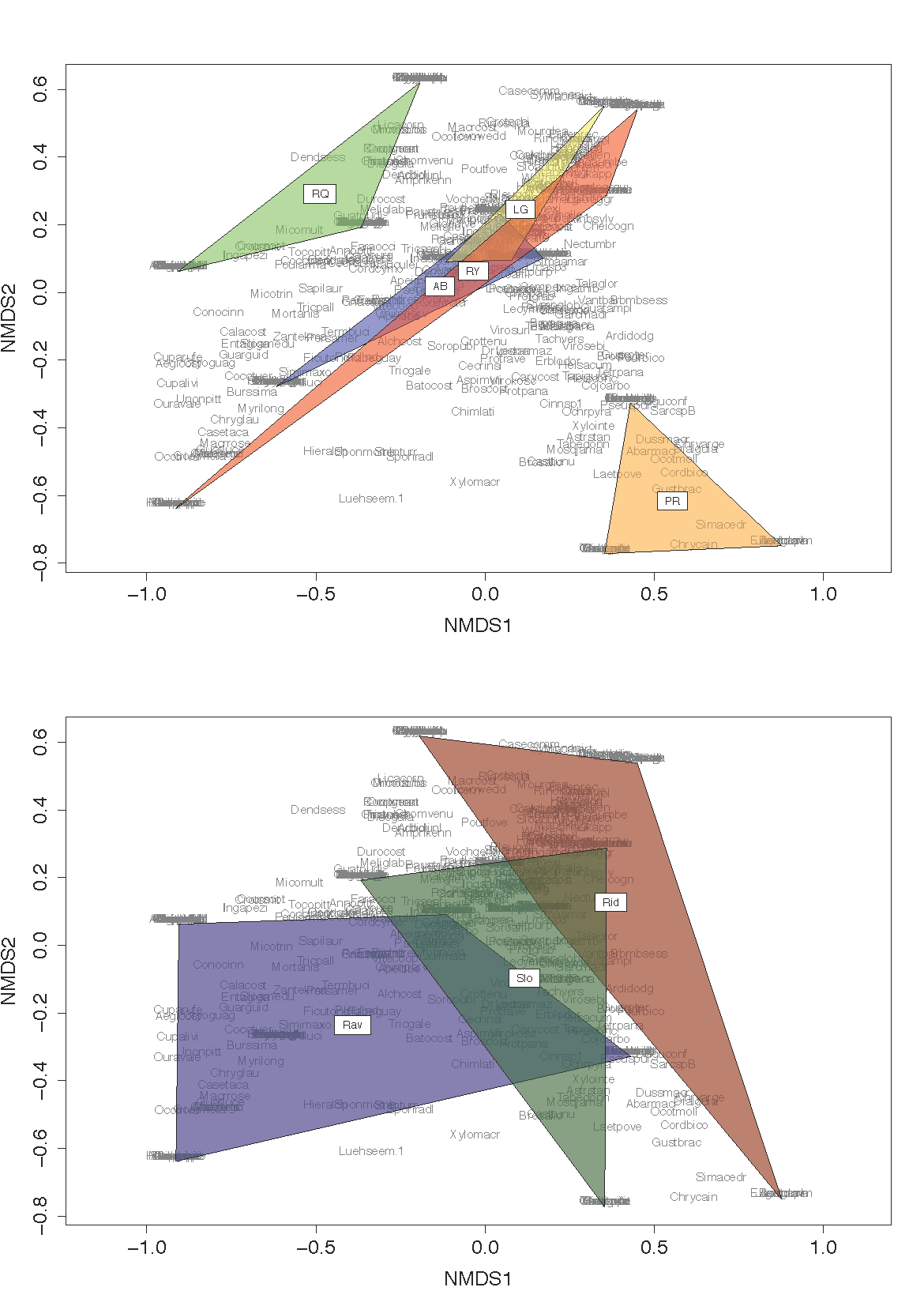
**

**Supplementary Figure S3**


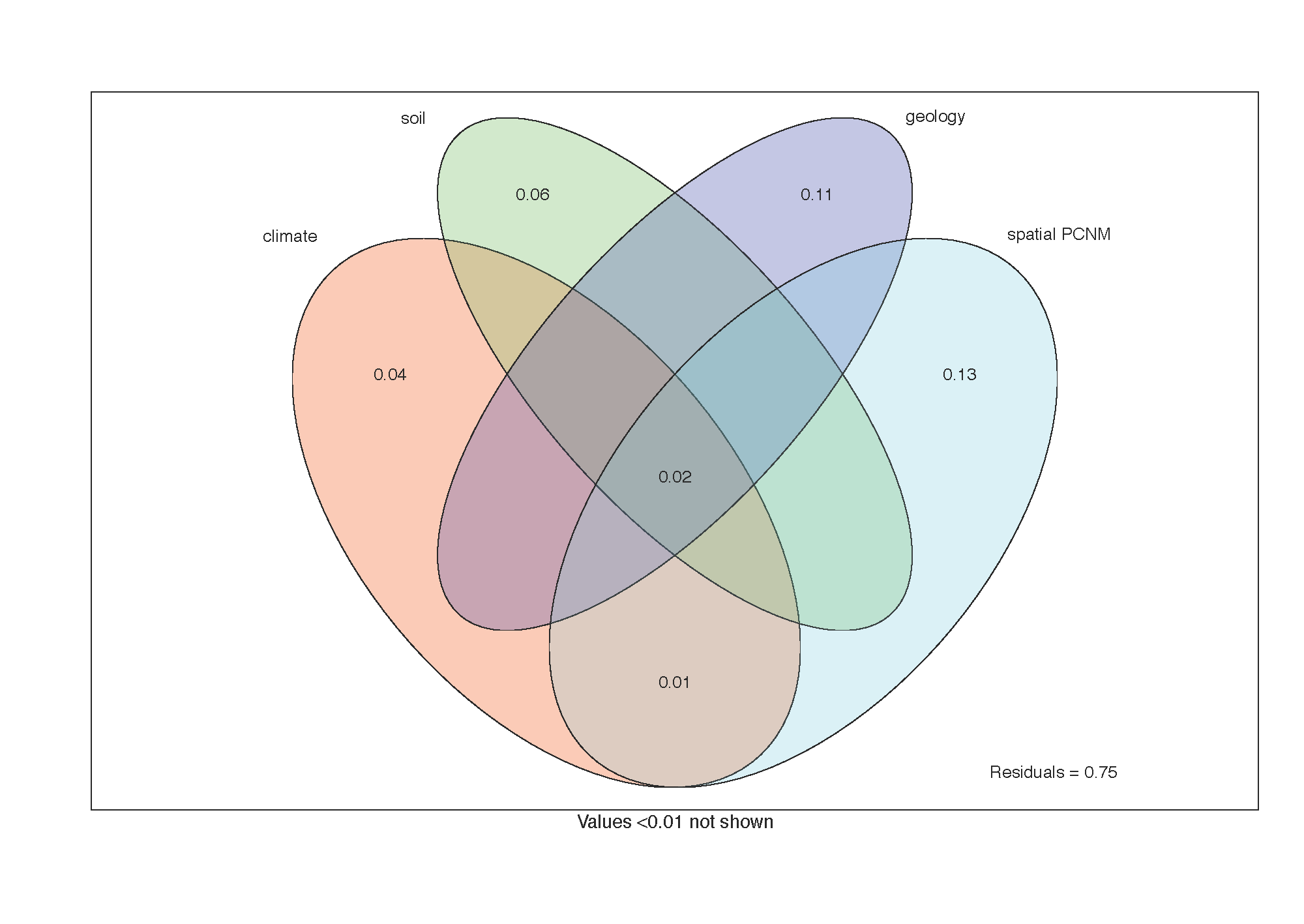


**Supplementary Figure S4**


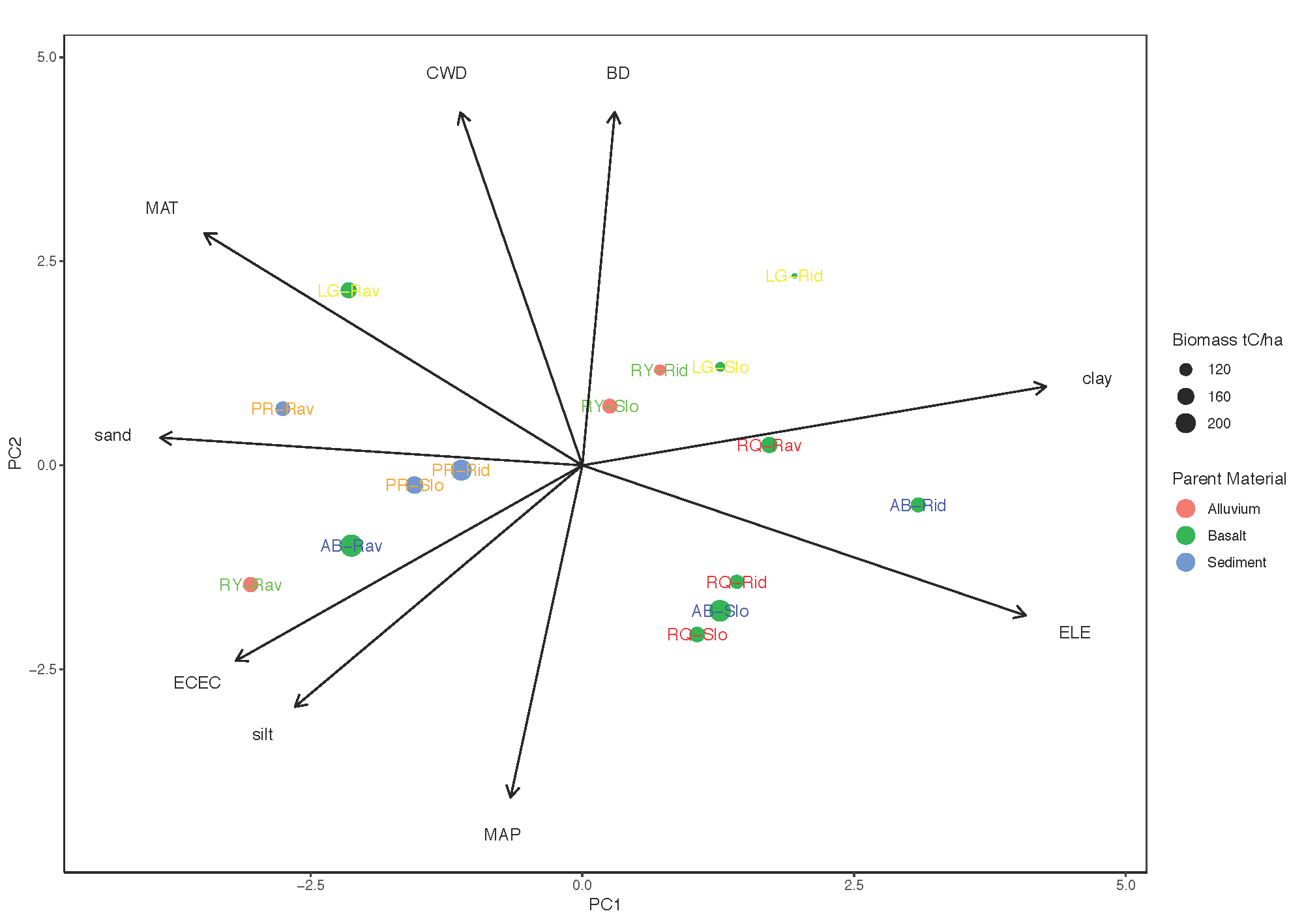


Supplementary Table S1

| **Parameter** | **Unit** | **LG** |  | **SE** | **RY** |  | **SE** | **AB** |  | **SE** | **RQ** |  | **SE** | **PR** |  | **SE** | **Ridge** |  | **SE** | **Slope** |  | **SE** | **Ravine** |  | **SE** |
| --- | --- | --- | --- | --- | --- | --- | --- | --- | --- | --- | --- | --- | --- | --- | --- | --- | --- | --- | --- | --- | --- | --- | --- | --- | --- |
| Latitude | decimal degrees | 8.68 | ± | 0.01 | 8.73 | ± | 0.00 | 8.71 | ± | 0.00 | 8.68 | ± | 0.00 | 8.41 | ± | 0.01 | 8.65 | ± | 0.06 | 8.65 | ± | 0.06 | 8.64 | ± | 0.06 |
| Longitude | decimal degrees | -83.2 | ± | 0.0 | -83.3 | ± | 0.0 | -83.5 | ± | 0.0 | -83.6 | ± | 0.0 | -83.3 | ± | 0.0 | -83.4 | ± | 0.1 | -83.4 | ± | 0.1 | -83.4 | ± | 0.1 |
| Elevation | meters | 253 | ± | 59 | 210 | ± | 40 | 315 | ± | 81 | 355 | ± | 63 | 115 | ± | 39 | 333 | ± | 50 | 270 | ± | 50 | 146 | ± | 29 |
| **Climatic variables** |  |  |  |  |  |  |  |  |  |  |  |  |  |  |  |  |  |  |  |  |  |  |  |  |  |
| MAT | degree Celsius | 25.6 | ± | 0.2 | 25.9 | ± | 0.0 | 24.7 | ± | 0.4 | 25.1 | ± | 0.2 | 26.4 | ± | 0.1 | 25.3 | ± | 0.4 | 25.4 | ± | 0.4 | 25.9 | ± | 0.2 |
| MATvar | degree Celsius | 63.1 | ± | 0.3 | 63.1 | ± | 0.0 | 64.8 | ± | 0.5 | 66.0 | ± | 0.2 | 70.0 | ± | 0.1 | 65.1 | ± | 1.3 | 65.2 | ± | 1.3 | 65.8 | ± | 1.3 |
| MAP | mm/yr | 4262 | ± | 51 | 4752 | ± | 0 | 4715 | ± | 74 | 4620 | ± | 221 | 4891 | ± | 116 | 4717 | ± | 138 | 4750 | ± | 106 | 4477 | ± | 115 |
| MAPvar | mm/yr | 45.9 | ± | 1.2 | 55.3 | ± | 0.0 | 53.5 | ± | 0.6 | 53.1 | ± | 1.9 | 57.6 | ± | 0.3 | 52.9 | ± | 2.3 | 53.1 | ± | 2.1 | 53.1 | ± | 1.9 |
| CWD | mm/yr | -96.6 | ± | 0.9 | -95.4 | ± | 1.9 | -194.1 | ± | 3.1 | -211.9 | ± | 6.2 | -158.6 | ± | 3.2 | -148.6 | ± | 23.2 | -148.7 | ± | 23.2 | -156.6 | ± | 26.2 |
| **Edaphic properties** |  |  |  |  |  |  |  |  |  |  |  |  |  |  |  |  |  |  |  |  |  |  |  |  |  |
| Water content | % | 48 | ± | 2 | 44 | ± | 2 | 40 | ± | 3 | 41 | ± | 4 | 40 | ± | 2 | 40 | ± | 2 | 46 | ± | 1 | 42 | ± | 3 |
| Sand content | % | 20 | ± | 8 | 17 | ± | 5 | 21 | ± | 10 | 11 | ± | 1 | 22 | ± | 6 | 12 | ± | 2 | 13 | ± | 1 | 29 | ± | 6 |
| Silt content | % | 35 | ± | 2 | 42 | ± | 5 | 35 | ± | 3 | 39 | ± | 2 | 40 | ± | 3 | 36 | ± | 3 | 38 | ± | 1 | 39 | ± | 3 |
| Clay content | % | 46 | ± | 10 | 41 | ± | 10 | 44 | ± | 12 | 51 | ± | 3 | 38 | ± | 5 | 51 | ± | 5 | 49 | ± | 1 | 31 | ± | 6 |
| pH | -log10[H+] | 4.1 | ± | 0.3 | 4.7 | ± | 0.3 | 4.7 | ± | 0.4 | 4.3 | ± | 0.2 | 4.9 | ± | 0.2 | 4.2 | ± | 0.1 | 4.6 | ± | 0.2 | 4.9 | ± | 0.3 |
| ECEC | meq | 35.8 | ± | 7.3 | 39.3 | ± | 8.2 | 46.7 | ± | 4.5 | 38.1 | ± | 7.9 | 56.5 | ± | 10.6 | 35.3 | ± | 3.9 | 45.3 | ± | 6.9 | 49.2 | ± | 7.1 |
| Carbon | % | 3.21 | ± | 0.51 | 5.56 | ± | 0.77 | 4.29 | ± | 0.16 | 4.01 | ± | 0.09 | 4.27 | ± | 1.06 | 4.94 | ± | 0.58 | 3.71 | ± | 0.31 | 4.16 | ± | 0.64 |
| Nitrogen | % | 0.25 | ± | 0.05 | 0.43 | ± | 0.06 | 0.38 | ± | 0.02 | 0.35 | ± | 0.01 | 0.38 | ± | 0.10 | 0.43 | ± | 0.05 | 0.33 | ± | 0.03 | 0.33 | ± | 0.05 |
| Phosphorus | g/kg | 0.04 | ± | 0.01 | 0.09 | ± | 0.03 | 0.08 | ± | 0.03 | 0.12 | ± | 0.02 | 0.08 | ± | 0.01 | 0.07 | ± | 0.01 | 0.07 | ± | 0.01 | 0.11 | ± | 0.02 |
| **Vegetation structure** |  |  |  |  |  |  |  |  |  |  |  |  |  |  |  |  |  |  |  |  |  |  |  |  |  |
| Stem number | n/ha | 592 | ± | 55 | 501 | ± | 68 | 520 | ± | 18 | 540 | ± | 48 | 432 | ± | 24 | 526 | ± | 51 | 549 | ± | 37 | 476 | ± | 27 |
| Stem basal area | m2/ha | 24.8 | ± | 2.2 | 26.1 | ± | 1.5 | 31.7 | ± | 2.1 | 27.5 | ± | 2.5 | 28.0 | ± | 1.4 | 25.9 | ± | 1.6 | 28.9 | ± | 1.8 | 28.0 | ± | 1.7 |
| Stem diameter | cm | 19.4 | ± | 0.0 | 21.6 | ± | 1.1 | 22.4 | ± | 0.7 | 21.0 | ± | 0.1 | 23.5 | ± | 0.6 | 21.7 | ± | 0.8 | 21.3 | ± | 0.7 | 21.8 | ± | 0.9 |
| Tree height | m | 44.4 | ± | 2.4 | 46.2 | ± | 5.0 | 55.3 | ± | 2.5 | 50.0 | ± | 1.4 | 45.2 | ± | 3.2 | 49.2 | ± | 2.8 | 49.1 | ± | 2.9 | 46.4 | ± | 3.1 |
| Leaf area index | m2/m2 | 8.0 | ± | 1.1 | 10.2 | ± | 0.8 | 9.5 | ± | 1.0 | 11.3 | ± | 0.6 | 10.8 | ± | 1.2 | 9.1 | ± | 1.1 | 11.1 | ± | 0.4 | 9.8 | ± | 0.7 |
| Wood density | g/cm3 | 0.51 | ± | 0.02 | 0.54 | ± | 0.03 | 0.53 | ± | 0.02 | 0.53 | ± | 0.03 | 0.57 | ± | 0.02 | 0.52 | ± | 0.02 | 0.53 | ± | 0.02 | 0.56 | ± | 0.01 |
| Wood biomass | t/ha | 224 | ± | 28 | 240 | ± | 11 | 370 | ± | 45 | 267 | ± | 7 | 297 | ± | 31 | 260 | ± | 29 | 291 | ± | 34 | 288 | ± | 32 |
| Root biomass | g/g | 237 | ± | 33 | 211 | ± | 55 | 266 | ± | 104 | 410 | ± | 90 | 292 | ± | 137 | 277 | ± | 81 | 317 | ± | 74 | 255 | ± | 60 |
| Carbon stock | tC/ha | 114 | ± | 18 | 129 | ± | 10 | 200 | ± | 31 | 141 | ± | 6 | 172 | ± | 23 | 136 | ± | 21 | 154 | ± | 21 | 163 | ± | 19 |
| **Species composition** |  |  |  |  |  |  |  |  |  |  |  |  |  |  |  |  |  |  |  |  |  |  |  |  |  |
| Species number | n/ha | 106 | ± | 2 | 126 | ± | 8 | 127 | ± | 7 | 94 | ± | 6 | 69 | ± | 9 | 104 | ± | 14 | 111 | ± | 12 | 99 | ± | 7 |
| Species diversity | Fisher's alpha | 38.0 | ± | 1.8 | 52.0 | ± | 2.4 | 54.2 | ± | 5.6 | 34.3 | ± | 2.4 | 23.4 | ± | 3.6 | 39.9 | ± | 7.2 | 43.1 | ± | 6.9 | 38.1 | ± | 3.6 |
| AM | % | 95.1 | ± | 0.9 | 92.6 | ± | 2.2 | 97.3 | ± | 0.5 | 96.4 | ± | 0.1 | 93.8 | ± | 3.2 | 94.6 | ± | 1.1 | 95.7 | ± | 1.4 | 94.8 | ± | 1.9 |
| EM | % | 4.7 | ± | 0.9 | 7.1 | ± | 2.1 | 2.2 | ± | 0.3 | 3.4 | ± | 0.2 | 6.1 | ± | 3.3 | 5.0 | ± | 1.2 | 3.9 | ± | 1.4 | 5.2 | ± | 1.9 |
| NM | % | 0.2 | ± | 0.1 | 0.2 | ± | 0.2 | 0.5 | ± | 0.2 | 0.2 | ± | 0.1 | 0.1 | ± | 0.1 | 0.4 | ± | 0.1 | 0.4 | ± | 0.1 | 0.0 | ± | 0.0 |
| Nfix | % | 5.1 | ± | 2.6 | 5.8 | ± | 0.6 | 4.3 | ± | 0.6 | 5.1 | ± | 0.2 | 3.5 | ± | 0.6 | 4.2 | ± | 1.0 | 4.1 | ± | 0.3 | 5.9 | ± | 1.1 |
| Palm | % | 25.3 | ± | 1.2 | 15.0 | ± | 6.1 | 11.5 | ± | 2.2 | 11.5 | ± | 4.9 | 2.4 | ± | 0.6 | 9.2 | ± | 4.2 | 15.9 | ± | 5.0 | 14.3 | ± | 3.6 |
| Liana | % | 1.2 | ± | 0.6 | 1.0 | ± | 0.3 | 2.2 | ± | 0.5 | 1.4 | ± | 0.1 | 0.9 | ± | 0.4 | 1.4 | ± | 0.5 | 1.0 | ± | 0.3 | 1.6 | ± | 0.2 |

Supplementary Table S2

| **Parameter** | **Forest region** | | | **Forest type** | | | **Geologic substrate** | | | **Soil type** | | |
| --- | --- | --- | --- | --- | --- | --- | --- | --- | --- | --- | --- | --- |
| fixed | region |  |  | site |  |  | parent material |  |  | soil type |  |  |
| random | site |  |  | region |  |  | region |  |  | region |  |  |
| Test statistic | F (4,8) | p-value | level | F (2,8) | p-value | level | F (2,2) | p-value | level | F (2,2) | p-value | level |
| **Climatic factors** |  |  |  |  |  |  |  |  |  |  |  |  |
| MAT | 18.9 | 0.00 | *** | 7.9 | 0.01 | * | 4.1 | 0.20 |  | 4.1 | 0.20 |  |
| MATvar | 229.6 | 0.00 | *** | 7.3 | 0.02 | * | 6.8 | 0.13 |  | 5.0 | 0.17 |  |
| MAP | 6.7 | 0.01 | * | 4.5 | 0.05 | . | 1.0 | 0.51 |  | 0.7 | 0.57 |  |
| MAPvar | 13.8 | 0.00 | ** | 0.0 | 0.99 |  | 1.1 | 0.47 |  | 0.6 | 0.62 |  |
| CWD | 431.9 | 0.00 | *** | 5.2 | 0.04 | * | 0.5 | 0.66 |  | 0.3 | 0.77 |  |
| **Edaphic properties** |  |  |  |  |  |  |  |  |  |  |  |  |
| Water content | 2.5 | 0.13 |  | 2.9 | 0.11 |  | 0.2 | 0.83 |  | 0.9 | 0.51 |  |
| Sand content | 1.1 | 0.41 |  | 8.0 | 0.01 | * | 0.4 | 0.73 |  | 1.1 | 0.48 |  |
| Silt content | 0.8 | 0.55 |  | 0.3 | 0.74 |  | 2.8 | 0.26 |  | 0.6 | 0.63 |  |
| Clay content | 0.5 | 0.71 |  | 4.8 | 0.04 | * | 3.1 | 0.24 |  | 1.1 | 0.48 |  |
| pH | 1.8 | 0.22 |  | 3.0 | 0.11 |  | 1.1 | 0.47 |  | 1.4 | 0.41 |  |
| ECEC | 1.2 | 0.37 |  | 1.5 | 0.28 |  | 3.4 | 0.23 |  | 42.7 | 0.02 | * |
| Carbon | 2.1 | 0.17 |  | 1.9 | 0.21 |  | 3.5 | 0.22 |  | 0.0 | 1.00 |  |
| Nitrogen | 1.7 | 0.24 |  | 2.1 | 0.19 |  | 1.0 | 0.50 |  | 0.1 | 0.92 |  |
| Phosphorus | 2.3 | 0.15 |  | 2.1 | 0.19 |  | 0.0 | 0.99 |  | 0.0 | 1.00 |  |
| **Vegetation structure** |  |  |  |  |  |  |  |  |  |  |  |  |
| Stem number | 1.6 | 0.27 |  | 1.1 | 0.39 |  | 3.9 | 0.21 |  | 2.2 | 0.31 |  |
| Stem basal area | 1.7 | 0.24 |  | 1.0 | 0.42 |  | 0.1 | 0.89 |  | 6.1 | 0.14 |  |
| Stem diameter | 4.8 | 0.03 | * | 0.3 | 0.74 |  | 1.2 | 0.46 |  | 2.8 | 0.27 |  |
| Total tree height | 1.8 | 0.22 |  | 0.4 | 0.70 |  | 0.4 | 0.73 |  | 3.9 | 0.20 |  |
| Leaf area index | 2.3 | 0.15 |  | 2.3 | 0.16 |  | 0.2 | 0.81 |  | 0.2 | 0.85 |  |
| Wood density | 1.4 | 0.31 |  | 2.6 | 0.14 |  | 4.4 | 0.18 |  | 3.6 | 0.22 |  |
| Aboveground biomass | 3.9 | 0.05 | * | 0.6 | 0.60 |  | 0.2 | 0.85 |  | 13.1 | 0.07 | . |
| Root biomass | 0.6 | 0.68 |  | 0.2 | 0.85 |  | 0.4 | 0.73 |  | 0.0 | 0.98 |  |
| Carbon stock | 2.9 | 0.09 | . | 0.7 | 0.52 |  | 0.2 | 0.81 |  | 12.4 | 0.07 | . |
| **Species composition** |  |  |  |  |  |  |  |  |  |  |  |  |
| Species number | 15.2 | 0.00 | *** | 1.7 | 0.24 |  | 7.2 | 0.12 |  | 3.4 | 0.23 |  |
| Species diversity | 13.6 | 0.00 | ** | 0.9 | 0.44 |  | 1.9 | 0.34 |  | 2.8 | 0.27 |  |
| AM | 0.9 | 0.50 |  | 0.5 | 0.60 |  | 68.4 | 0.01 | * | 0.4 | 0.72 |  |
| EM | 1.0 | 0.47 |  | 0.5 | 0.62 |  | 33.4 | 0.03 | * | 0.5 | 0.66 |  |
| NM | 1.9 | 0.21 |  | 6.4 | 0.02 | * | 0.4 | 0.74 |  | 32.3 | 0.03 | * |
| Nfix | 4.3 | 0.04 | * | 2.6 | 0.13 |  | 20.4 | 0.05 | * | 0.5 | 0.66 |  |
| Palm | 6.5 | 0.01 | * | 2.2 | 0.18 |  | 1.1 | 0.47 |  | 1.6 | 0.38 |  |
| Liana | 1.9 | 0.20 |  | 0.2 | 0.79 |  | 1.1 | 0.49 |  | 2.9 | 0.26 |  |

Supplementary Table S3

| **Row Labels** | **AB-Rid** | **AB-Rav** | **AB-Slo** | **LG-Rid** | **LG-Rav** | **LG-Slo** | **PR-Rid** | **PR-Rav** | **PR-Slo** | **RY-Rid** | **RY-Rav** | **RY-Slo** | **RQ-Rid** | **RQ-Rav** | **RQ-Slo** | **Total** |
| --- | --- | --- | --- | --- | --- | --- | --- | --- | --- | --- | --- | --- | --- | --- | --- | --- |
| **Iriartea deltoidea** | 80 | 39 | 76 | 53 | 40 | 148 |  |  |  | 49 | 1 | 80 | 76 | 15 | 65 | **722** |
| **Welfia regia** |  |  |  | 52 | 116 | 21 |  |  |  |  | 14 | 57 | 3 | 1 |  | **264** |
| **Compsoneura excelsa** | 5 | 60 | 37 | 3 | 5 | 11 |  | 5 | 48 |  | 23 | 25 | 1 | 29 | 9 | **261** |
| **Otoba novogranatensis** | 28 | 13 | 21 | 36 | 1 | 15 | 9 | 1 | 8 | 4 |  | 7 | 46 | 13 | 44 | **246** |
| **Symphonia globulifera** | 6 | 21 | 14 | 3 | 18 | 12 | 24 | 23 | 12 | 1 | 14 | 23 | 6 | 23 | 20 | **220** |
| **Carapa nicaraguensis** | 3 | 15 | 8 | 19 | 6 | 22 | 19 |  |  | 3 | 17 | 18 | 6 | 24 | 16 | **176** |
| **Tapirira guianensis** | 1 | 7 | 4 | 1 | 8 | 7 | 24 | 41 | 33 | 3 | 9 |  | 1 | 7 | 12 | **158** |
| **Mabea occidentalis** |  | 6 |  |  | 59 | 33 |  |  |  |  | 35 | 23 |  |  |  | **156** |
| **Tetrathylacium macrophyllum** | 18 | 4 | 28 | 23 | 6 | 13 | 7 |  | 3 | 11 |  | 11 | 12 | 4 | 15 | **155** |
| **Qualea paraensis** |  | 3 | 5 |  | 34 |  | 20 |  |  |  | 41 | 40 |  | 8 |  | **151** |
| **Perebea hispidula** | 4 | 24 | 2 | 6 | 2 | 4 | 17 | 8 | 4 | 2 | 18 | 8 | 1 | 6 | 11 | **117** |
| **Socratea exorrhiza** |  | 4 |  | 24 | 14 | 7 | 10 | 15 | 3 | 1 | 11 | 6 | 5 | 3 | 2 | **105** |
| **Pausandra trianae** | 6 | 14 |  | 12 | 3 | 2 |  |  |  |  |  |  | 1 | 35 | 31 | **104** |
| **Tetragastris panamensis** | 2 | 6 | 2 | 2 | 2 | 8 | 25 | 31 | 10 |  | 5 | 6 |  |  | 4 | **103** |
| **Grand Total** | 555 | 507 | 497 | 507 | 696 | 573 | 474 | 390 | 433 | 395 | 479 | 628 | 448 | 559 | 612 | **7753** |

Supplementary Table S4

| **Row Labels** | **Site** | **Type** | **AMF** | **EMF** | **NM** | **tree** | **palm** | **liana** | **Nfix** | **Total** |
| --- | --- | --- | --- | --- | --- | --- | --- | --- | --- | --- |
| **AB-Rid** | AB | Ridge | 548 | 6 | 1 | 466 | 83 | 5 | 17 | **555** |
| **AB-Rav** | AB | Ravine | 491 | 11 | 5 | 460 | 43 | 3 | 20 | **507** |
| **AB-Slo** | AB | Slope | 481 | 13 | 3 | 415 | 78 | 4 | 15 | **497** |
| **LG-Rid** | LG | Ridge | 501 | 6 |  | 368 | 139 |  | 26 | **507** |
| **LG-Rav** | LG | Ravine | 652 | 42 | 2 | 507 | 187 |  | 7 | **696** |
| **LG-Slo** | LG | Slope | 566 | 5 | 2 | 387 | 180 | 5 | 8 | **573** |
| **PR-Rid** | P | Ridge | 417 | 57 |  | 461 | 11 | 2 | 9 | **474** |
| **PR-Rav** | P | Ravine | 372 | 16 | 2 | 375 | 15 |  | 4 | **390** |
| **PR-Slo** | P | Slope | 432 | 1 |  | 425 | 7 | 1 | 3 | **433** |
| **RY-Rid** | R | Ridge | 386 | 9 |  | 337 | 57 | 1 |  | **395** |
| **RY-Rav** | R | Ravine | 425 | 53 | 1 | 440 | 35 | 2 |  | **479** |
| **RY-Slo** | R | Slope | 572 | 52 | 4 | 432 | 195 |  |  | **628** |
| **RQ-Rid** | RQ | Ridge | 431 | 17 |  | 347 | 99 | 2 | 19 | **448** |
| **RQ-Rav** | RQ | Ravine | 540 | 17 | 2 | 532 | 23 | 4 | 19 | **559** |
| **RQ-Slo** | RQ | Slope | 596 | 13 | 3 | 540 | 69 | 3 | 6 | **612** |
|  |  |  |  |  |  |  |  |  |  | **7753** |
